# The coupling of Phanerozoic continental weathering and marine phosphorus cycle

**DOI:** 10.1038/s41598-020-62816-z

**Published:** 2020-04-02

**Authors:** Ruimin Wang, Xianguo Lang, Weiming Ding, Yarong Liu, Tianzheng Huang, Wenbo Tang, Bing Shen

**Affiliations:** 1 Key Laboratory of Orogenic Belts and Crustal Evolution, MOE & School of Earth and Space Sciences, Peking University, Beijing, 100871 China; 2 State Key Laboratory of Oil and Gas Reservoir Geology and Exploitation & Institute of Sedimentary Geology, Chengdu University of Technology, Chengdu, 610059 China; 3 School of Mathematical & Statistical Sciences, Arizona State University, Tempe, AZ 85287 USA

**Keywords:** Biogeochemistry, Ocean sciences

## Abstract

Organic matter production and decomposition primarily modulate the atmospheric O_2_ and CO_2_ levels. The long term marine primary productivity is controlled by the terrestrial input of phosphorus (P), while the marine P cycle would also affect organic matter production. In the past 540 million years, the evolution of terrestrial system, e.g. colonization of continents by vascular land plants in late Paleozoic, would certainly affect terrestrial P input into the ocean, which in turn might have impacted the marine primary productivity and organic carbon burial. However, it remains unclear how the marine P cycle would respond to the change of terrestrial system. Here we reconstruct the secular variations of terrestrial P input and biological utilization of seawater P in Phanerozoic. Our study indicates that riverine dissolved P input and marine P biological utilization (i.e. the fraction of P being buried as organophosphorus) are inversely correlated, suggesting the coupling of continental P input and marine P cycle. We propose an increase of P input would elevate surface ocean productivity, which in turn enhances marine iron redox cycle. Active Fe redox cycle favors the scavenging of seawater P through FeOOH absorption and authigenic phosphate formation in sediments, and accordingly reduces the bioavailability of seawater P. The negative feedback of marine P cycle to terrestrial P input would keep a relatively constant organic carbon burial, limiting the variations of surface Earth temperature and atmospheric O_2_ level.

## Introduction

Although the biodiversity goes up and down and the animal evolution has been frequently punctuated by mass extinction and the subsequent recovery^1^, the Earth System remained habitable for animals in the past 540 million years (Myr). The sustained habitability was warranted by the limited variations of the temperature and the redox condition of the surface Earth. It is widely accepted that the global temperature is mainly controlled by the pCO_2_ level in the atmosphere^2^, while the surface Earth redox condition is determined by the atmosphere O_2_ content^3^. In spite of the long-term oscillation between the icehouse and greenhouse/hothouse climatic conditions^4^, devastating extreme climate events, such as the global glaciations, have never occurred after the Cryogenian (~720-635 Ma) snowball Earth events^5^. Nor has the atmospheric pO_2_ level ever dropped below the threshold of 10% present atmospheric level (PAL) for animal breath^6^. It is proposed that the atmospheric O_2_ level might have increased from 10–40% PAL in the early Cambrian to the modern level in late Paleozoic, and showed limited variation in the past 250 million years^6,7^, although oceanic anoxia could be common and widespread in certain geological ages^8^.

The atmospheric pCO_2_ and pO_2_ levels are modulated by the global carbon cycle, in which organic matter production and destruction in both terrestrial and marine systems are the core processes^9^. The terrestrial carbon cycle might be negligible before the diversification of vascular land plant in late Paleozoic^10,11^, but the marine carbon cycle has been the key component of global carbon cycle throughout the Earth’s history. As such, the secular variation of marine primary productivity would provide a key constraint on the evolution of atmospheric pCO_2_ and pO_2_ levels.

The marine primary productivity is dominated in the upper most 200 m of the ocean (the euphotic zone), and is controlled by the availability of macronutrients, including phosphorus (P), nitrogen (N) and silicon (Si), and micronutrients, such as iron (Fe), zinc (Zn) etc.^12^. Unlike the bio-available N that can be synthesized by diazotrophs (nitrogen-fixing cyanobacteria) by using atmospheric N_2_ gas as ingredient^13^, the P mainly derives from terrestrial input^14,15^. It has long been held that the riverine P input primarily controls the marine primary productivity in the geological time scale^15,16^, placing the upper bound of surface ocean primary productivity and confining the largest magnitudes of CO_2_ drawdown or O_2_ rise in the atmosphere.

On the other hand, marine P cycle might impact the short-term primary productivity at local or regional scales^15,16^. Substantial amount of seawater P would either be either converted to authigenic phosphate or absorbed by iron oxyhydroxides (FeOOH)^15,17^, reducing the bio-availability of seawater P. In the modern abyssal sediments, authigenic phosphate precipitation and absorption by FeOOH would account for more than 80% of P burial^15^. Thus, the long-term marine primary productivity is controlled by both riverine P influx and marine P cycle. In this study, we reconstruct the Phanerozoic secular variations of terrestrial input of dissolved P (P_in_) and the fraction of organophosphorus burial with respect to the total marine phosphorus burial (R_p_) by using the seawater strontium isotope and the carbonate carbon isotope data.

## Model Descriptions

### Data acquisition

The Phanerozoic Strontium isotope $((\frac{{}^{87}Sr}{{}^{86}Sr}){}_{sw})$ and carbonate carbon isotope (δ^13^C_carb_) data were collected from chapters in the book: *The Geologic Time Scale 2012*^18,19^. Based on the variation of $(\frac{{}^{87}Sr}{{}^{86}Sr}){}_{sw}$ and δ^13^C_carb_ curves, Phanerozoic (541 Ma-present) was divided into 65 intervals, each of which is defined by distinct trends of ${\left(\frac{{}^{87}Sr}{{}^{86}Sr}\right)}_{sw}$ and/or δ^13^C_carb_ as compared with adjacent intervals (Table S1). The time intervals can be classified as the long (>3 Myr) and short (= or <3 Myr) duration intervals, reflecting the long-term and short-term variations in $(\frac{{}^{87}Sr}{{}^{86}Sr}){}_{sw}$ and δ^13^C_carb_, respectively. Totally 41 long duration intervals and 24 short duration intervals were identified (Table S1), and the total range of the long duration intervals (493 Myr) accounts for 91% of Phanerozoic (541 Myr).

### Quantifying the riverine influx of dissolved phosphorus

River water is the only major sources of both Sr and P in the ocean, and the riverine influxes are directly controlled by the continental weathering. Riverine P input includes dissolved and particulate P, while Sr is mainly transported in its dissolved form^15,18,20–22^. Overall, particulate P would have limited effects on the atmospheric pO_2_ and pCO_2_ levels for the following reasons. Firstly, detrital particulate inorganic P (e.g. unweathered apatite from igneous rocks or fossil authigenic phosphate from sedimentary rocks) is geochemically inactive, and thus cannot involve in the biogeochemical reactions in the ocean. Secondly, although particulate organic P could be mobilized by the degradation of associated organic component and might have different C: P ratios from the Redfield ratio of 106: 1^23^, organic matter remineralization releases CO_2_ and consumes oxidants, offsetting, though not necessarily counterbalancing, the CO_2_ consumption and O_2_ emission in photosynthesis. Thirdly, the iron-bound P from continental input could be remobilized by reduction of ferric Fe, which is associated with organic matter remineralization and releases of ferrous Fe (Fe^2+^), resulting in the CO_2_ emission and the reduction of oxidative state of the ocean^14,15,20^. Thus, remobilization of particulate P tends to cancel the effect of organic matter production and thus have limited impact on the atmospheric pO_2_ and pCO_2_ levels. As such, primary productivity fueled by riverine dissolved P would directly impact the pCO_2_ and pO_2_ levels of the atmosphere.

To reconstruct the terrestrial dissolved P input, we approach with $(\frac{{}^{87}Sr}{{}^{86}Sr}){}_{sw}$, which documents the mixing between hydrothermal and riverine inputs^21,22^. Assuming the marine Sr budget (${{\rm{M}}}_{Sr}^{0}$) remains a constant, i.e. the riverine and hydrothermal Sr inputs are counterbalanced by carbonate precipitation, the isotope mass balance of marine Sr cycle can be expressed as:

1 $$\frac{d\left(\frac{{}^{87}Sr}{{}^{86}Sr}\right){}_{sw}}{dt}=\frac{{F}_{Sr}^{r}\left(\frac{{}^{87}Sr}{{}^{86}Sr}\right){}_{r}+{F}_{Sr}^{hy}\left(\frac{{}^{87}Sr}{{}^{86}Sr}\right){}_{hy}-({F}_{Sr}^{r}+{F}_{Sr}^{hy})\left(\frac{{}^{87}Sr}{{}^{86}Sr}\right){}_{carb}}{{M}_{Sr}^{0}}$$

where ${F}_{Sr}^{i}$ is the Sr flux of source or sink i, and the subscripts r and hy represent the riverine and hydrothermal inputs, respectively. Because carbonate precipitation is the only major sink of seawater Sr, and Sr in carbonate minerals records the seawater Sr isotopic composition, i.e. $\left(\frac{{}^{87}Sr}{{}^{86}Sr}\right){}_{sw}=\left(\frac{{}^{87}Sr}{{}^{86}Sr}\right){}_{carb}$^21^

By solving the differential Eq. 1, riverine influx of Sr (${F}_{Sr}^{r}$) can be calculated by the following equation:

2 $$\frac{{F}_{Sr}^{r}[\left(\frac{{}^{87}Sr}{{}^{86}Sr}\right){}_{r}-\left(\frac{{}^{87}Sr}{{}^{86}Sr}\right){}_{sw2}]+{F}_{Sr}^{hy}[\left(\frac{{}^{87}Sr}{{}^{86}Sr}\right){}_{hy}-\left(\frac{{}^{87}Sr}{{}^{86}Sr}\right){}_{sw2}]}{{F}_{Sr}^{r}[\left(\frac{{}^{87}Sr}{{}^{86}Sr}\right){}_{r}-\left(\frac{{}^{87}Sr}{{}^{86}Sr}\right){}_{sw1}]+{F}_{Sr}^{hy}[\left(\frac{{}^{87}Sr}{{}^{86}Sr}\right){}_{hy}-\left(\frac{{}^{87}Sr}{{}^{86}Sr}\right){}_{sw1}]}={e}^{\frac{({F}_{Sr}^{r}+{F}_{Sr}^{hy})(t1-t2)}{{M}_{Sr}^{0}}}$$

where subscript sw1 and sw2 are the seawater compositions at the beginning and the end of the time interval. Because the riverine dissolved P is mainly derived from weathering of continent rocks^15^, the relationship between the riverine dissolved P (${F}_{P}^{in}$) and Sr inputs (${F}_{Sr}^{r}$) can be expressed by the following equation:

3 $${F}_{P}^{in}=\gamma \cdot {f}_{dSr}\cdot {f}_{dP}\cdot {F}_{Sr}^{r}$$

where *γ* is the ratio between P and Sr concentrations of the weathered continental rocks, *f*_*dSr*_ and *f*_*dp*_ are the fraction of dissolved Sr and P fluxes in the total terrestrial Sr and P inputs.

### Quantifying the marine phosphorus cycle

Seawater dissolved P can be buried with organic carbon as organophosphorus, as authigenic phosphate, and by adsorption onto FeOOH, i.e. Fe-bound phosphorus^14,15,17^. The amount of organophosphorus burial is proportional to the quantity of organic matter burial, which can be represented by the following equation:

4 $${F}_{org}={P}_{in}\times {R}_{P}\times RED$$

where *F*_*org*_ is the amount of organic carbon burial, ${R}_{p}=\frac{{P}_{org}}{{P}_{in}}$ represents the proportion of organophosphorus burial with respect to the total dissolved P input from continental weathering, and RED is the Redfield ratio of marine primary productivity with a theoretical C: P (molar ratio) of 106:1^23–25^.

The mass and carbon isotope balances can be expressed as:

5 $$\Delta ={\delta }_{DIC}-{\delta }_{org}$$

6 $$\frac{d{\delta }_{DIC}}{dt}=\frac{{F}_{C}^{in}{\delta }_{in}-{F}_{C}^{in}{\delta }_{DIC}+\Delta {F}_{org}}{{M}_{C}^{0}+({F}_{C}^{in}-{F}_{pre}-{F}_{org})\,t}$$

where Δ is the biological C isotope fractionation in photosynthesis, ${F}_{C}^{in}$ and ${\delta }_{in}$ are the flux and isotopic composition of DIC from continental weathering, ${F}_{pre}$ is the flux of marine carbonate precipitation, ${\delta }_{org}^{\,}$ is the isotopic composition of buried organic carbon, and ${\delta }_{DIC}$ is the isotopic composition of the seawater DIC that is recorded in marine carbonate, i.e. ${\delta }^{13}{C}_{DIC}={\delta }^{13}{C}_{carb}$. *M*_0_^C^ is the size of dissolved inorganic carbon (DIC) pool in the ocean.

The terrestrial DIC input is also related to the continental weathering, which is reflected by the mass ratio between the dissolution of silicate rock and carbonate rock^26^. The mass and isotopic balance of continent weathering can be expressed as:

7 $${F}_{C}^{in}={F}_{CarW}+{F}_{SilW}$$

8 $${\delta }_{in}=\frac{{F}_{CarW}{\delta }_{CarW}+{F}_{SilW}{\delta }_{SilW}}{{F}_{CarW}+{F}_{SilW}}$$

where the subscript *CarW* and *SilW* represent carbonate rock and silicate rock weathering, respectively.

To link the riverine DIC and dissolved Sr influxes, we define the following relationship:

9 $${F}_{C}^{in}=A{F}_{Sr}^{r}$$

where A is the coefficient between mass of silicate rock weathering and riverine Sr input, and can be determined by the modern values (Table S2). Combining Eqs. 4–9, *R*_*p*_ can be calculated by the following equation:

10 $${R}_{p}=\frac{A{F}_{Sr}^{r}\times {\delta }_{DIC2}-A{F}_{Sr}^{r}\times {\delta }_{DIC1}{e}^{-\frac{A{F}_{Sr}^{r}}{\Delta M}(Ln({M}_{0}+t\varDelta M)-Ln({M}_{0}))}}{\Delta \,(1-{e}^{-\frac{A{F}_{Sr}^{r}}{\Delta M}(Ln({M}_{0}+t\Delta M)-Ln({M}_{0}))})\,{F}_{P}^{in}\,RED}-\frac{{F}_{C}^{in}{\delta }_{in}}{\Delta {F}_{P}^{in}RED}$$

### Parameter setting

The Phanerozoic seawater Sr isotope and carbonate carbon isotope data are tabulated in Table S1, and the assigned values of all parameters are listed in Table S2. $\left(\frac{{}^{87}Sr}{{}^{86}Sr}\right){}_{r}$, $\left(\frac{{}^{87}Sr}{{}^{86}Sr}\right){}_{hy}$, and ${M}_{Sr}^{0}$ are 0.7119, 0.7035, and 1.12 × 10^19^ g, respectively^21^. Hydrothermal Sr influx (${F}_{Sr}^{hy}$) is estimated from the oceanic crust production rate (assuming the linear correlation), given the modern ${F}_{Sr}^{hy}$ of 1 Tg/yr and the production rate of oceanic crust of 2.9 km^2^/yr^27,28^. ${\delta }_{CarW}$ and ${\delta }_{SilW}$ are assigned as −2.5‰ and −5‰^29^. The ratio between ${F}_{C}^{in}$ and ${F}_{Sr}^{r}$ (A) is calculated from the modern values of 89^30^. The biological fractionation of organic carbon production (Δ) is 25‰^31^.

We further assume the normal distribution of the carbonate rock weathering (${F}_{CarW}$) N (130 Tg/yr, 30^2^)^30^. The size of seawater DIC could also fluctuation. We assume that normal distribution of the rate of seawater DIC pool variation (△M) with the sigma of 39 × 10^18^ g, i.e. double the DIC pool within the integrated interval, N (0,39^2^) (10^18^ g). The parameter γ, the P/Sr ratio of weathered continental rocks is dependent on the lithology of exposed rocks. Especially, the exposure of large igneous province (LIP) in continents, e.g. the Siberian trap with the area of 2.1 million km^2^ ^32^, would el evate the P/Sr ratio in certain geological interval. We assume the probability of LIP eruption with the exposure area of Siberian trap is 0.01, and the normal dis tribution of γ with the mean value of the upper continental crust composition (P: 655 ppm, Sr: 320 ppm)^33^.

We run Monte Caro simulations with repeated assigning the unconfined parameters (*F*_*CarW*_ ΔM, and γ) 1000 times, and the mean value and standard deviation are calculated from 1000 iterations. The model calculate the modern ${F}_{Sr}^{r}$, P_in_ and R_p_ of 2.9 Tg/yr, 1.2 Tg/yr and 0.15, respectively, consistent with the observed values^15,21^.

### Secular variation of terrestrial input of dissolved P and marine P cycle

**Figure 1:**
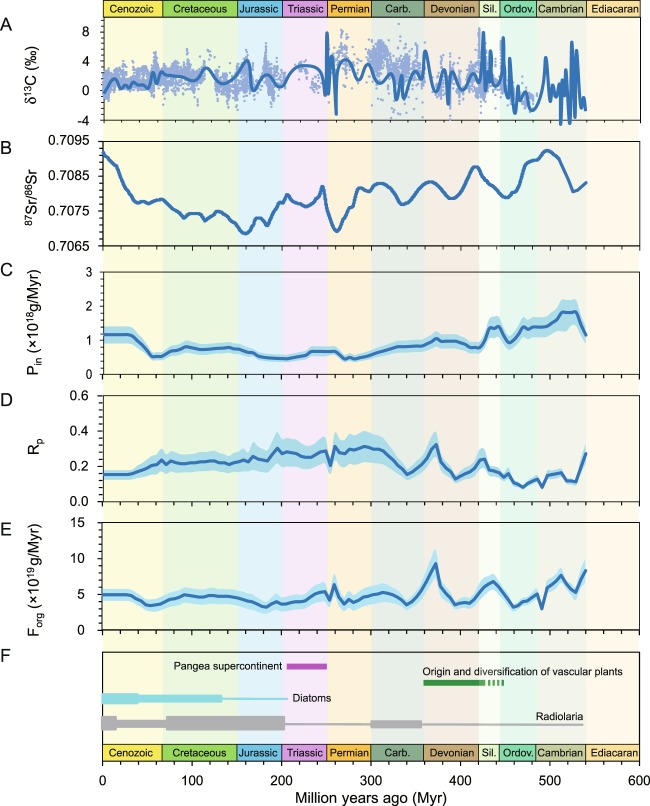
(**A**) Secular variation of carbonate carbon isotopes. Data are from Saltzman and Thomas^19^ and Prokoph, *et al*.^64^. (**B**) Secular variation of seawater strontium isotopes. Data are from McArthur, *et al*.^18^. (**C**) Secular variation of riverine influx of dissolved phosphate (P_in_). (**D**) Secular variation of fraction of organophosphorus burial with respect to total riverine influx of dissolved P (R_p_). (**E**) Secular variation of mass of organic carbon burial (F_org_). (**F**) Evolutionary trend of silica-secreting organisms (diatom and radiolarian), showing the stepwise diversification in Mesozoic^62^, and the width of the box represents the diversity; also marked the time lines of vascular land plants evolution^11^ and assembly and breakup of Pangea^63^. The solid lines in **C**,**D**,**E** are the mean values of Monte Caro simulation with 1000 iterations, while the shadowed regions bracket the 2 times of standard deviations (i.e. 95% confidence interval) of 1000 iterations.

The secular variations of terrestrial input of dissolved P (P_in_) and fraction of organophosphorus burial (R_p_) can be calculated from the Phanerozoic carbonate carbon and strontium isotope data (Fig. 1A,B, Table S1). The modeling results indicate that P_in_ and R_p_ display the long-term opposite trend (Fig. 1C,D). P_in_ shows a general decreasing trend in Paleozoic, remains at a low value in Mesozoic, and increases in Cenozoic. In contrast, R_P_ displays an increasing trend in Paleozoic followed by a high-value interval in Mesozoic, and declines in Cenozoic (Fig. 1C,D).

Nevertheless, both P_in_ and R_P_ show more complex variations in the shorter time intervals. P_in_ displays a general decreasing trend from ~2 Tg/yr in the early Cambrian to ~1 Tg/yr in late Ordovician (540-470 Ma), while R_P_ remains at a low values of ~0.1 within this time interval. In the subsequent 40 million years (470-420 Ma), P_in_ first increases to ~1.5 Tg/yr in late Ordovician, remains at the high value in early Silurian (445-425 Ma), and decreases to ~0.8 Tg/yr in the Silurian-Devonian boundary. In contrast, R_p_ illustrates a steady increase from 0.1 to 0.2 within 40 million years (470-430 Ma) and a sharp decline to 0.1 in the following 30 million years (430-400 Ma). In the following 150 million years (400-250 Ma), there is a continuous decline of P_in_ from ~1 Tg/yr to ~0.5 Tg/yr, but R_p_ shows more complex variations. R_p_ increases sharply from 0.1 to 0.3 from 400 Ma to 370 Ma and then decreases to 0.1 in the following 30 million years (370-340 Ma). In the last 40 million years of Carboniferous (340-300 Ma), R_p_ returns to the highest value of 0.3 and remained in the plateau throughout Permian until the Permian-Triassic transition that is characterized by a rapid decline to 0.2. In the whole Mesozoic (250-65 Ma), P_in_ slightly oscillates around ~0.5 Tg/yr, while R_p_ shows a slight decrease from 0.3 to 0.2. P_in_ increases to the modern value of 1.2 Tg/yr in the first 30 million years of Cenozoic (66-35 Ma), while R_p_ displays an opposite trend. In the last 35 million years, both P_in_ and R_P_ remains at the modern values (Fig. 1C,D).

### Coupling of terrestrial input of dissolved P and marine P cycle

**Figure 2:**
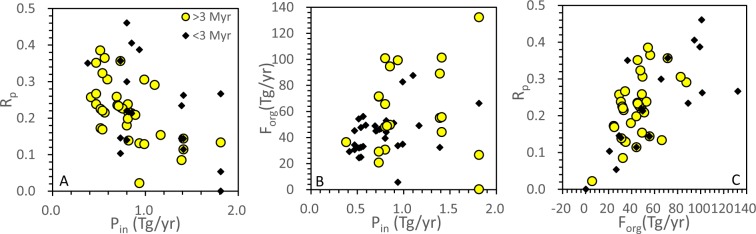
(**A**) Crossplot showing the negative correlation between P_in_ and R_p_. (**B**) Crossplot showing the absence of correlation between F_org_ and P_in_. (**C**) Crossplot showing the absence of correlation between F_org_ and R_p_ for the long duration intervals (yellow circles), and a crude correlation for the short duration intervals (dark dots). The yellow circles represent the intervals greater than 3 million years (long duration) while the dark dots indicate intervals less than 3 million years (short duration).

The negative correlation between P_in_ and R_p_ implies the coupling of terrestrial P input and marine P cycle (Figs. 1C,D, 2A). An increase of P_in_ would associate with the lower efficiency of biological P utilization in the ocean (Fig. 2A). In contrast, when the riverine influx of dissolved P is low, more P is buried as organophosphorus, implying less fraction of dissolved P being sequestered as authigenic phosphate or iron-bound P^15^.

The riverine input of dissolved P determines the upper bound of marine primary productivity, and confines the maximum burial of organic carbon. However, not all seawater P would be buried with organic matter, instead, substantial amount of seawater P would be eventually sequestered as authigenic phosphate or iron-bound P^15,17^, reducing the bioavailability of seawater P. In the modern abyssal sediment where detrital P deposition is negligible, authigenic phosphate and iron-bound P account for ~70% and ~15% of P burial, respectively^15^.

The iron (Fe) redox cycle plays the key role in the P biogeochemical cycle between seawater and sediment^15,17^. In oxic conditions, ferrous Fe (Fe^2+^) is thermodynamically unstable and would be spontaneously oxidized to ferric Fe (Fe^3+^). In the oxic seawater with near neutral pH of 8–8.2, ferric Fe would precipitate as iron oxyhydroxides (FeOOH), during which seawater P would be absorbed by FeOOH. The degree of P absorption, measured by the P to Fe molar ratio [(P/Fe)_100_] of iron oxides/oxyhydroxides deposits, is controlled by the concentrations of dissolved P and Si in seawater, the latter of which would compete with P for the absorption sites in FeOOH^34,35^. Precipitation of FeOOH particles shuttles seawater P into sediments. Further reduction of FeOOH by iron reducing microbes (IRM) in sediment liberates absorbed P into porewater, resulting in the precipitation of authigenic phosphate by reacting with Ca^2+^ and HCO_3_^−^ in sediment porewater^17,36^. On the other hand, Fe^2+^ could diffuse back into seawater, i.e. the benthic iron flux^37,38^, where ferrous Fe oxidation and FeOOH precipitation would continue shuttling seawater P into sediments^34^. If FeOOH is not reduced in sediment, e.g. due to the depletion of organic matter, preservation of FeOOH would result in the Fe-bound P burial. Therefore, the amount of iron-bound P burial is determined by the availability of reactive Fe that involves in the redox cycle between seawater and sediment, while authigenic phosphate burial is controlled by both the number of Fe redox cycling and the amount of cycled reactive Fe.

The Fe redox cycle is mainly driven by the microbial iron reduction (MIR) that uses reactive ferric Fe (e.g. Fe_2_O_3_, FeOOH) as electron acceptor to oxidize organic matter to bicarbonate^36^. Thus, active Fe redox cycle requires sufficient supplies of organic matter. The Fe redox cycle in the ocean and continental weathering are related in that, high riverine flux of dissolved P would stimulate high surface ocean productivity^15^, which in turn enhances organic matter supply for MIR. On the other hand, high productivity also lowers O_2_ fugacity at the sediment-water interface (SWI), enhancing the benthic Fe^2+^ flux from sediment porewater to seawater^37–40^. Therefore, high P_in_ favors repeated Fe reduction-oxidation cycles between seawater and sediment, enhancing the shuttling of seawater P into sediment.

The negative correlation between P_in_ and R_P_ also implies the negative feedbacks imposed by marine P cycle as a response to the change of riverine input of dissolved P (Fig. 1), which limits the variation of organic carbon burial. As shown in Fig. 1, though δ^13^C_carb_ displays dramatic oscillation in Phanerozoic (Fig. 1A), the amount of organic carbon burial (F_org_) remains more or less constant throughout Phanerozoic (Fig. 1E).

Although generally negatively correlated, P_in_ and R_p_ do not show exact opposite trends (Fig. 1C,D). For example, in late Ordovician and Silurian, P_in_ first decreases from 1.5 to 1 Tg/yr, then followed by a sharp increase to  2 Tg/yr and decrease within 40 million years. In contrast, R_p_ displays a steady increase from 0.08 to 0.25(Fig. 1C,D). The reason for the sudden increase of P_in_ might be related to the closure of Iapetus ocean and the formation of Caledonian Orogeny^41^, the latter of which might have substantially enhanced weathering input of P, while the steady increase of R_p_ might reflect, (1) the diversifications of phytoplanktons and zooplanktons in the Great Ordovician Biodiversification Event, which might have enhanced the efficiency of particulate organic carbon burial^42–44^, and (2) the ocean oxygenation in Silurian as evidenced by the global formation of marine red beds depositions^45,46^.

In addition, a decrease of P_in_ in late Cretaceous (100-65 Ma) does not associate with an increase of R_P_. Instead, R_P_ remains nearly invariant throughout Cretaceous. The invariant R_P_ might be attributed to, in the context of super-greenhouse climatic condition in Cretaceous, the widespread oceanic anoxia^47,48^, under which condition the Fe redox cycle might be insensitive to the surface ocean productivity. It should be also noted that the flourish of silica-secreting organisms, such as diatom and radiolarian (Fig. 1F), might have lowered the seawater Si concentration^49^, enhanced P absorption onto FeOOH particles^35^, and accordingly promoted the efficiency of P delivery by FeOOH shuttle. However, it remains unclear how change of seawater Si concentration could affect the marine P cycle in Cretaceous.

### Effect of vascular land plant diversification on the riverine P input and marine P cycle

The modeling result indicates a decrease of P_in_ in late Paleozoic (Fig. 1C), when vascular land plants diversified and the forest began to form^10,11^. It is widely accepted that the biological weathering is more efficient than inorganic chemical weathering that uses CO_2_ as reactant^10,50^. With the presence of land plant, weathering intensity could be substantially enhanced in several ways. First of all, the decomposition of organic matter in soils would generate more corrosive organic acid that accelerates the reaction rate, while the respiration of plant roots also elevates CO_2_ concentration in soils, further enhancing mineral dissolution^51^. Moreover, plant roots would split rocks apart, favoring water percolation and increasing the reactive surface within rock interior. The enhanced continental weathering intensity in late Paleozoic is supported by the widespread sedimentary bauxite depositions since late Devonian^52^. Therefore, it is predicted that riverine influx of P would be higher, when continents were colonized by vascular land plants.

In fact, the riverine influx of P not only depends on the rate of chemical weathering, but also is a function of physical erosion^53,54^. Rapid erosion would reduce the reaction time of chemical weathering. The erosion rate would be smaller in well-vegetated continents. and is sensitive to regional/local precipitation and temperature. We speculate that low P_in_ in late Paleozoic might be related to the amalgamation of supercontinents Pangea (Fig. 1C,F), during which the weakened hydrological cycles in the ocean-continent system would result in an expansion of drought area in the continent interior^55,56^. Low P_in_ in Carboniferous and Permian might also relate to the global cooling during the late Paleozoic ice age^57^, since weathering rate is temperature dependent^58^. For the same reason, an increase of P_in_ in late Mesozoic might be attributed to an elevated continental hydrological cycle during the breakup of Pangea (Fig. 1C,F)^56^ as well as the greenhouse/hothouse climatic conditions^4^.

Diversification of vascular land plant in late Devonian to early Carboniferous (400-340 Ma) is also witnessed the significant decoupling of P_in_ and R_P_. R_p_ displays a sharp increase from ~0.1 to 0.3 followed by a decrease to 0.08, while P_in_ shows a slight decrease from 1.2 to 0.8 Tg/yr (Fig. 1C,D). The reason for the rapid change of R_P_ is unknown. We speculate that the increase of R_p_ in mid-Devonian might be attributed to, e.g. oxygenation in atmosphere due to the formation of forest^6,11^.

### The long-term and short-term coupling of continent-ocean system

We propose that P_in_ reflects the long-term change of continental weathering, while R_p_ is driven by the short-term marine P cycles. Organic carbon burial as well as δ^13^C_carb_ was controlled by both the long-term and short-term processes. Therefore, δ^13^C_carb_ does not show any correlation with either P_in_ or R_p_ (Fig. 1A,C,D). In addition, there is no correlation between F_org_ and P_in_ (yellow circles in Fig. 2B) or between F_org_ and R_p_ (yellow circles in Fig. 2C) for the long duration intervals (>3 Myr). In contrast, the short-term (<3 Myr) δ^13^C_carb_ excursions might be mainly controlled by the change of R_p_. This argument is well supported by the crude correlation between F_org_ and R_p_ in the short duration intervals (black dots in Fig. 2C).

Some short duration δ^13^C_carb_ excursions, such as, the negative δ^13^C_carb_ excursion between 525-523 Ma (the DICE event)^59^, cannot be explained by the canonical carbon cycle model^29^. The DICE event is characterized by a negative R_p_ value (Table S1). The negative R_p_ may indicate additional P supply from a non-terrestrial source, such as remineralization of a dissolved organic carbon (DOC) pool in the deep ocean^60^.

### Closing remarks

Our study indicates that the continent and ocean systems have been tightly coupled throughout the Phanerozoic. Though the continent system could be perturbed by, e.g., assembly and breakup of supercontinents^56^, eruption of large igneous provinces^61^, or evolution of terrestrial ecosystem^10^, resulting in a dramatic change of riverine input of dissolved P. Such perturbations could be mitigated by marine P cycle. More specifically, the coupled continent and ocean systems would limit the variation of organic carbon burial, which plays the key role in modulating atmospheric pCO_2_ and pO_2_ levels^29^, and accordingly the global temperature and the Earth's surface redox conditions. Thus, the coupling of continental P weathering and marine P cycle warrants the long-term stability and habitability of the Earth system in the past 540 million years, paving the way for the evolution of intelligent human beings.

## Supplementary information

Supplementary information.

## Data Availability

All data is available in the main text or the supplementary materials.
