# Supplementary material for: The coupling of Phanerozoic continental weathering and marine phosphorus cycle
Source: Sci Rep. 2020 Apr 2;10:5794. doi: 10.1038/s41598-020-62816-z (PMC7118102; doi:10.1038/s41598-020-62816-z)
Supplement: Supplementary file 1 — Supplementary information. [file 41598_2020_62816_MOESM1_ESM.pdf]

The coupling of Phanerozoic continental weathering and marine phosphorus cycle

Ruimin Wang[a], Xianguo Lang[b], Weiming Ding[a], Yarong Liu[a], Tianzheng Huang[a], Wenbo Tang[c], Bing Shen[a,*]

supplementary information include：

Table 1： Carbon and Strontium isotopes and the modeling outputs
Table 2： Parameters used in the simulation

Table.S1. Carbon and Strontium isotopes and the modeling outputs

t_1 (Ma)	t_2 (Ma)	$\delta^{13}C_1$ (‰)	$\delta^{13}C_2$ (‰)	$^{87}Sr/^{86}Sr_{r1}$	$^{87}Sr/^{86}Sr_{r2}$	F_{hy} (Tg/yr)	F^{Sr}_{r} (Tg/yr)	F_{org}_mean (Tg/yr)	F_{org}_std (Tg/yr)	F^{P}_{in}_mean (Tg/yr)	F^{P}_{in}_std (Tg/yr)	R_p_mean	R_p_std
540	531	-4.00	5.00	0.7083	0.7081	2.27	2.74	87.67	9.00	1.10	0.11	0.291	0.030
531	528	5.00	-2.00					26.63	0.60	1.81	0.19	0.054	0.001
528	525	-2.00	6.00					132.31	10.20	1.81	0.19	0.267	0.021
525	523	6.00	-4.00	0.7081	0.7091	2.27	4.51	0.21	1.80	1.81	0.19	0.000	0.004
523	518	-4.00	1.00					66.26	4.20	1.81	0.19	0.134	0.009
518	497	1.00	1.00					66.26	4.20	1.81	0.19	0.134	0.009
497	495	1.00	4.00					89.11	7.80	1.39	0.14	0.234	0.021
495	494	4.00	1.00	0.7091	0.7087	2.13	3.46	55.04	4.22	1.39	0.14	0.145	0.011
494	465	1.00	-1.00					32.38	1.80	1.39	0.14	0.085	0.005
465	463	-1.00	1.00					55.07	4.20	1.39	0.14	0.145	0.011
463	459	1.00	-3.00					5.68	0.60	0.94	0.10	0.022	0.002
459	453	-3.00	0.00	0.7087	0.7079	2.13	2.33	33.69	3.00	0.94	0.10	0.131	0.012
453	446	0.00	0.00					33.69	3.00	0.94	0.10	0.131	0.012
446	445	0.00	7.00					99.26	11.28	0.94	0.10	0.387	0.044
445	443	7.00	1.00					55.60	4.20	1.41	0.15	0.144	0.011
443	434	1.00	1.00					55.60	4.20	1.41	0.15	0.144	0.011
434	433	1.00	5.00	0.7079	0.7087	2.13	3.51	101.38	8.98	1.41	0.15	0.263	0.023
433	431	5.00	0.00					44.17	3.00	1.41	0.15	0.114	0.008
431	425	0.00	0.00					44.17	3.00	1.41	0.15	0.114	0.008
425	424	0.00	8.00					100.86	12.41	0.80	0.08	0.461	0.057
424	421	8.00	0.00					30.67	3.00	0.80	0.08	0.140	0.014
421	419	0.00	4.00	0.7087	0.7078	1.94	1.99	65.61	7.80	0.80	0.08	0.300	0.036
419	416	4.00	0.00					30.67	3.00	0.80	0.08	0.140	0.014
416	414	0.00	2.00					48.14	5.40	0.80	0.08	0.220	0.025
414	393	2.00	1.00					39.41	4.20	0.80	0.08	0.180	0.019
393	368	1.00	0.00	0.7078	0.7082	1.94	2.46	34.85	3.00	0.99	0.10	0.129	0.011
368	360	0.00	5.00					82.69	9.00	0.99	0.10	0.306	0.033
360	359	5.00	2.00					49.65	5.47	0.85	0.09	0.213	0.023
359	357	2.00	7.00	0.7082	0.7079	1.94	2.12	94.59	11.40	0.85	0.09	0.406	0.049
357	355	7.00	2.00					49.76	5.40	0.85	0.09	0.213	0.023
355	338	2.00	2.00					49.76	5.40	0.85	0.09	0.213	0.023
338	333	2.00	0.00					31.12	3.00	0.82	0.08	0.139	0.013
333	332	0.00	2.00	0.7079	0.7083	1.56	2.04	48.84	5.35	0.82	0.08	0.218	0.024
332	322	2.00	2.00					48.76	5.40	0.82	0.08	0.217	0.024

322	302	2.00	2.00	0.7083	0.7082	1.56	2.03	48.64	5.40	0.82	0.08	0.218	0.024
302	293	2.00	4.00	0.7082	0.7075	1.56	1.40	56.18	7.80	0.56	0.06	0.365	0.051
293	289	4.00	1.00					33.12	4.20	0.56	0.06	0.215	0.027
289	272	1.00	4.00	0.7075	0.7073	1.56	1.28	54.26	7.80	0.51	0.05	0.385	0.055
272	260	4.00	1.00	0.7073	0.7069	1.56	1.03	29.18	4.20	0.41	0.04	0.258	0.037
260	255	1.00	5.00					71.33	9.00	0.73	0.08	0.356	0.045
255	252	5.00	0.00					29.16	3.00	0.73	0.08	0.146	0.015
252	250	0.00	5.00	0.7069	0.7080	1.56	1.82	71.33	9.00	0.73	0.08	0.356	0.045
250	248	5.00	-1.00					20.73	1.80	0.73	0.08	0.104	0.009
248	247	-1.00	5.00					71.60	8.83	0.73	0.08	0.358	0.044
247	236	2.00	3.00	0.7080	0.7076	1.56	1.47	49.49	6.60	0.59	0.06	0.306	0.041
236	216	3.00	2.50	0.7076	0.7079	1.56	1.72	48.91	6.00	0.69	0.07	0.259	0.032
216	201	2.50	1.00					30.67	4.20	0.47	0.05	0.238	0.033
201	189	1.00	3.00	0.7079	0.7071	1.56	1.17	45.23	6.60	0.47	0.05	0.351	0.051
189	182	3.00	1.50					34.31	4.80	0.47	0.05	0.267	0.037
182	176	1.50	1.00	0.7071	0.7073	1.56	1.29	31.95	4.20	0.52	0.05	0.225	0.030
176	166	1.00	0.00	0.7073	0.7073	1.56		24.46	3.00	0.52	0.05	0.172	0.021
166	164	0.00	3.00	0.7073	0.7068	1.56	0.95	36.43	6.58	0.38	0.04	0.351	0.063
164	146	3.00	2.00					48.51	5.40	0.81	0.08	0.218	0.024
146	130	1.50	1.50	0.7068	0.7075	2.22	2.02	44.12	4.80	0.81	0.08	0.199	0.022
130	126	1.50	2.50					52.91	6.00	0.81	0.08	0.238	0.027
125	114	2.50	2.00	0.7075	0.7072	2.22	1.74	45.03	5.40	0.70	0.07	0.235	0.028
114	108	2.00	2.00	0.7072	0.7073	2.22	1.84	46.28	5.40	0.74	0.08	0.229	0.027
108	96	2.00	2.00	0.7073	0.7074	2.22	1.88	46.77	5.40	0.76	0.08	0.226	0.026
96	90	2.00	2.00	0.7074	0.7073	2.22	1.77	45.41	5.40	0.71	0.07	0.233	0.028
90	66	2.00	2.00	0.7073	0.7077	2.22	2.22	51.00	5.40	0.89	0.09	0.209	0.022
66	62	2.00	1.00					32.48	4.20	0.54	0.06	0.220	0.029
62	58	1.00	3.00	0.7077	0.7077	1.37	1.34	47.64	6.60	0.54	0.06	0.323	0.045
58	52	3.00	0.00					24.90	3.00	0.54	0.06	0.169	0.020
52	35	0.00	1.00					32.48	4.20	0.54	0.06	0.220	0.029
35	0	1.00	0.00	0.7077	0.7092	1.37	2.90	49.10	4.20	1.17	0.12	0.154	0.013

Table.S2. Parameters used in the simulation

parameters	values	unit	reference
Mo^{0}_{Sr}	1.12E+19	g	21
Mo^{0}_{C}	3.90E+19	g	30
$(^{87}Sr/^{86}Sr)r$	0.712		21
$(^{87}Sr/^{86}Sr)hy$	0.704		21
Fcarb	$N(130,30^2)$	Tg/yr	30
δ_{sil}	-5.00	‰	29
δ_{carb}	-2.50	‰	29
△	25.00	‰	31
RED	106		23-25
Sr（crust）	320	ppm	33
P（crust）	$N(655,130^2)$	ppm	2,32
f_{dp}	0.08		15,21
A	89		30
